# Network Pharmacology and Transcriptomic Sequencing Analyses Reveal the Molecular Mechanism of *Sanguisorba officinalis* Against Colorectal Cancer

**DOI:** 10.3389/fonc.2022.807718

**Published:** 2022-05-12

**Authors:** Weijia Zhang, Shuyi Sang, Chang Peng, George Q. Li, Ling Ou, Zhong Feng, Yuanjing Zou, Yuemei Yuan, Meicun Yao

**Affiliations:** ^1^ School of Pharmaceutical Sciences, Sun Yat-sen University, Guangzhou, China; ^2^ School of Pharmaceutical Sciences (Shenzhen), Sun Yat-sen University, Shenzhen, China; ^3^ Institute of Natural Products and Metabolomics, Chengdu University of Traditional Chinese Medicine, Chengdu, China; ^4^ School of Ecology, Sun Yat-sen University, Guangzhou, China

**Keywords:** colorectal cancer, *Sanguisorba officinalis*, network pharmacology, transcriptomic sequencing, PI3K–Akt pathway, MAPK pathway

## Abstract

**Background:**

Colorectal cancer (CRC) is the most common malignant cancer worldwide. *Sanguisorba officinalis* has been shown to have anti-inflammatory, anti-bacterial, antioxidant, and anti-tumor effects, while its molecular mechanism against CRC remains unclear. The aim of this study is to explore the underlying mechanism of *S. officinalis* against CRC cell lines using network pharmacology and transcriptomic sequencing methods.

**Method:**

Firstly, the active ingredients and potential targets of *S. officinalis* against CRC were screened from databases. Secondly, the networks of ingredient–target, ingredient–target–CRC and protein–protein interaction were constructed. Gene Ontology and Kyoto Encyclopedia of Genes and Genomes pathway enrichment analyses of network pharmacology and transcriptomic sequencing were performed. Finally, the effect of *S. officinalis* against CRC was verified by *in vitro* experiments.

**Results:**

In total, 14 active ingredients and 273 potential targets against CRC were identified in *S. officinalis* by network pharmacology. PI3K–Akt, HIF-1, and MAPK signaling pathways related to cell proliferation were regulated by *S. officinalis* in enrichment analyses and transcriptomic sequencing. *In vitro*, *S. officinalis* inhibited the proliferation and migration of CRC cells and arrested the cell cycle at the G0–G1 phase. The western blot showed that *S. officinalis* downregulated the expression of p-PI3K, p-Akt, HIF-1A, VEGFA, cyclin D1, c-Myc, and p-MAPK proteins in CRC cells.

**Conclusion:**

In conclusion, network pharmacology and transcriptomic sequencing analyses, in combination with *in vitro* studies, have been successfully applied to study the underlying mechanism of *S. officinalis* against CRC cells. Our results demonstrate that *S. officinalis* suppresses the proliferation, survival, and migration of CRC cells through regulating the PI3K–Akt, HIF-1, and MAPK signaling pathways.

## Introduction

Colorectal cancer (CRC) is a kind of cancer in the alimentary canal which ranks as the third in morbidity and mortality in the world (1). In 2020, CRC accounts for 10% of global cancer incidences and 9.4% of cancer deaths (2). Although the current therapeutic strategies have been improved, such as surgery, chemotherapy, targeted therapy, immunotherapy, or radiotherapy strategies, CRC still results in difficult treatment or poor prognosis for most patients because of its strong metastasis—for example, drug resistance and side effects associated with the use of chemotherapeutic agents lead to unsatisfactory clinical outcomes.

In recent years, traditional Chinese medicine (TCM), consisting of various active ingredients, has been studied against cancer considering its unique advantages of good efficacy and minimal side effects (3). *Sanguisorba officinalis* (known as Di Yu in Chinese), a member of Rosaceae family, exhibits a wide range of pharmacological activities, such as hemostatic, anti-inflammatory, anti-allergy, anti-bacterial, antioxidant, hypoglycemic, neuroprotective, and anticancer effects (4, 5). The latest studies found that *S. officinalis* exerted its therapeutic effects on hepatocellular carcinoma through interfering in cancer cell proliferation and survival *via* the EGFR/MAPK and EGFR/PI3K/AKT/NFκB signaling pathways (6). We have previously reported that *S. officinalis* synergistically enhanced 5-fluorouracil cytotoxicity in CRC cell lines (RKO and HCT116) by activating a reactive oxygen species-mediated and mitochondria caspase-dependent apoptotic pathway (7).

However, a systematic study with latest network pharmacology approaches on the molecular mechanism of *S. officinalis* against CRC is still lacking. Network pharmacology analyzes the links among drugs, targets, and diseases to demonstrate the synergistic effects of TCM on multiple targets and pathways (8). Transcriptomic research demonstrates gene functions and structures from the whole level and reveals the molecular mechanism of drug actions in diseases (9). Combining these two modern biological analysis methods helps to clarify the molecular mechanism of TCM. Therefore, the aim of this study is to explore the underlying mechanism of *S. officinalis* against CRC cell lines using network pharmacology and transcriptomic sequencing methods.

## Materials and Methods

### Screening of the Active Ingredients in *S. officinalis*

The potential chemical ingredients in *S. officinalis* were acquired from the Traditional Chinese Medicine Systems Pharmacology Database (TCMSP, http://tcmsp-e.com), Traditional Chinese Medicine Integrated Database (TCMID, http://www.megabionet.org/tcmid/), and references (5, 10). The active ingredients were selected according to absorption, distribution, metabolism, and excretion (ADME) principle, and the screening criteria were oral bioavailability (OB) >30% and drug likeness (DL) >0.18. The molecular structures were acquired from the PubChem database (https://pubchem.ncbi.nlm.nih.gov/).

### Identification of Potential Targets Related to CRC

The putative protein targets of active ingredients were accessed from TCMSP and Swiss Target Prediction database (STP, http://www.swisstargetprediction.ch/). The UniProt database (https://www.uniprot.org/) was applied to change the protein targets’ names to their corresponding gene symbols. The GeneCards database (https://www.genecards.org/), Online Mendelian Inheritance in Man database (OMIM, https://omim.org/), DrugBank online database (https://go.drugbank.com/) and Therapeutic Target Database (TTD, http://db.idrblab.net/ttd/) were searched to identify potential targets related to CRC by using keywords such as “colorectal cancer”, “colon carcinoma”, or “rectum carcinoma”.

### Construction of Active Ingredient–Disease–Target Network

The active ingredient–target network was constructed by Cytoscape, version 3.7.1 software (https://cytoscape.org/). The nodes represent ingredients or targets, and the edges reflect the interaction between nodes. The bioinformatics (http://www.bioinformatics.com.cn/) platform was performed to draw a Venn diagram of common target numbers of ingredient–disease. Cytoscape3.7.1 software was applied to construct the active ingredient–CRC-target network. The degree value represents the significance of the target.

### Construction of Protein–Protein Interaction Network

The protein–protein interaction (PPI) network of targets of the active ingredients against CRC were conducted by STRING software (http://string-db.org), with a correlation degree greater than 0.4 as the confidence score. Then, the nodes and score information were combined into the Cytoscape3.7.1 software for analysis. Degree, betweenness centrality, and closeness centrality were three parameters used to assess the topological features of nodes in the network. Among them, the size and the color of nodes both reflect the degree values.

### Gene Ontology and Kyoto Encyclopedia of Genes and Genomes Pathway Enrichment Analyses

The Metascape platform (http://metascape.org/) was used for Gene Ontology (GO) and Kyoto Encyclopedia of Genes and Genomes (KEGG) pathway enrichment analyses of common targets. The results of GO and KEGG pathway enrichment analyses were visualized on the bioinformatics platform. Cytoscape3.7.1 software was used to construct the target–pathway network. The nodes represent targets or signaling pathways, and the edges present the interaction between nodes.

### Chemicals and Reagents

3-(4,5-Dimethylthiazol-2-yl)-2,5-diphenyltetrazolium bromide (MTT) was purchased from MP (Solon, OH, USA). Dulbecco’s modified Eagle’s medium (DMEM), penicillin–streptomycin, phosphate-buffered saline (PBS), fetal bovine serum (FBS), Pierce BCA protein assay kit, and Super Signal™ West Pico Chemiluminescent Substrate kit were obtained from Thermo Fisher Scientific (Waltham, MA, USA). Calcein/propidium iodide (PI) kit, RIPA buffer, crystal violet, 4% paraformaldehyde, and PI/RNase kit were from Beyotime Biotechnology (Jiangsu, China). Trizol buffer was purchased from Invitrogen (Carlsbad, CA, USA). The primary antibodies against cyclin D1, c-Myc, PI3K, P-PI3K, Akt, P-Akt, HIF-1A, VEGFA, MAPK, and P-MAPK were purchased from Affinity (Cincinnati, OH, USA), and β-actin and GAPDH were from Cell Signaling Technology (Beverly, MA, USA). The secondary antibodies were obtained from Millipore Corporation (Temecula, CA, USA).

### Preparation of *S. officinalis* Aqueous Extracts

*S. officinalis* (Jiangsu, China, no. 201902) was authenticated by Prof. Depo Yang (Sun Yat-sen University). The dried rhizome of *S. officinalis* was boiled and refluxed with ultrapure water for 1 h at each time (total of 3 times). After filtration, the extracted solution was evaporated, then lyophilized to dried powder, and stored at -20°C before use.

### Cell Lines and Culture

RKO and HCT-15 cells were used in the following experiments, which were kindly gifted by Professor Huangliang Liu from Sixth Affiliated Hospital, Sun Yat-Sen University, China. All cell lines were cultured in DMEM containing 10% FBS and 1% penicillin–streptomycin under a humidified incubator with 5% CO_2_ at 37°C.

### Cell Proliferation Assay

Cell proliferation of CRC cells was determined by MTT assay. The cells were seeded in 96-well plates (5,000 cells/well). After 24 h of incubation, the cells were treated with different concentrations of *S. officinalis* extract (31.25 to 500 μg/ml) for 24 or 48 h. Then, 20 μl of 5 mg/ml MTT buffer was added to each well and incubated for 4 h at 37°C. The absorbance at 570 nm was measured on a microplate reader (Molecular Devices, Flex Station 3, Sunnyvale, CA, USA).

### Cell Viability and Cytotoxicity Assay

CRC cells were seeded into 96-well plates at a density of 5 × 10^3^ cells per well, incubated for 24 h, and then exposed to various concentrations of *S. officinalis* extract (0, 50, and 100 μg/ml) for 48 h. Then, 100 μl of calcein/PI solution was added to each well for 30 min of incubation at 37°C in the dark. Then, the cells were observed and photographed under a fluorescence microscope (Olympus, Tokyo, Japan).

A total of 1 ×10^5^ CRC cells were seeded into each well of 6-well plates and exposed to different concentrations of *S. officinalis* extract (0, 50, and 100 μg/ml) for 48 h. Then, the cells were washed twice by PBS, fixed in 4% paraformaldehyde for 20 min, and stained with crystal violet.

### Cell Cycle Assay

After 24 h of treatment with *S. officinalis* extract (0, 50, and 100 μg/ml), the cells were harvested and incubated with PI/RNase solution (Beyotime, China). Then, cellular samples were immediately analyzed on flow cytometry (Temecula, CA, USA), see the details in a previous study (11).

### Wound Healing Assay

Cells were incubated in 6-well plates with 100% confluence and then scratched using a sterile pipette tip on the cell monolayer. The cells were washed 3 times with PBS, and *S. officinalis* extract was added to each well (0, 50, and 100 μg/ml). After 0, 12, and 24 h of incubation, the wound areas were observed under a light microscope (Olympus, Tokyo, Japan). The wound areas were measured by ImageJ software and normalized with the control group.

### Total RNA Extraction and mRNA Library Construction

The transcriptomics analysis was accomplished by our collaborator, Beijing Genomics Institute (BGI, Shenzhen, China). The cells were incubated in 6-well plates and exposed to *S. officinalis* extract (100 μg/ml), and total RNA was extracted according to the manual’s instruction and quantified using a Nano Drop and Agilent 2100 bioanalyzer (Thermo Fisher Scientific, MA, USA). The mRNA library was constructed on a BGISEG500 platform according to the BGI protocol (https://pan.genomics.cn/ucdisk/s/BFFr6j).

### Analysis of miRNA Sequencing Data

The sequencing data was analyzed according to the BGI protocol (https://pan.genomics.cn/ucdisk/s/RzEr6b), and differently expressed genes (DEGs) were screened [false discovery rate (FDR) ≤0.001, |log_2_FC| ≥1]. Then, GO and KEGG enrichment analyses of DEGs were performed by Phyper. The terms and pathways are significant when *Q* ≤0.05.

### Western Blotting Assay

Cells were treated in the same way as described in the RT-q PCR assay. The total protein was extracted from the cells, and then the concentration was determined. Protein samples were loaded in gels and transferred to polyvinylidene fluoride films. Then, the films were incubated with primary antibodies and secondary antibodies. Finally, the films were imaged by Automatic Chemiluminescence Image Analysis System (Tanon 5200, Shanghai, China), see the details in a previous study (12).

### Statistical Analysis

The obtained data were expressed as mean ± SD (n = 3). The statistical analysis was performed by t-test by GraphPad Prism 8.0 software, and p <0.05 was considered to be significantly different.

## Results

### Identification and Validation of Active Ingredients in *S. officinalis*

Through TCMSP, TCMID, and literatures, over 100 compounds were found in *S. officinalis*. After using ADME screening parameters (OB >30% and DL >0.18), a total of 14 candidate compounds were identified as active ingredients of *S. officinalis* (**Table 1**). According to the previous studies, we found that some compounds were detected in *S. officinalis* by UPLC-MS (**Supplementary Figure S1**).

**Table 1:** Active ingredients in *S. officinalis*.

Number	Active ingredients	Molecule ID	Molecular weight	Oral bioavailability (%)	Drug likeness	Structure
1	Mairin	MOL000211	456.78	55.38	0.78	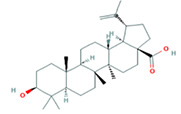
2	Beta-sitosterol	MOL000358	414.79	36.91	0.75	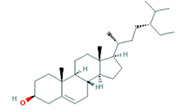
3	Kaempferol	MOL000422	286.25	41.88	0.24	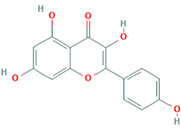
4	Alexandrin_qt	MOL005399	414.79	36.91	0.75	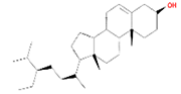
5	Methyl-2,3,6-tri-O-galloyl-β-D-glucopyranoside	MOL005853	654.68	44.95	0.67	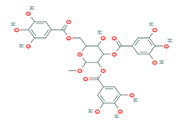
6	3,7,8-Tri-O-methylellagic acid	MOL005858	344.29	37.54	0.57	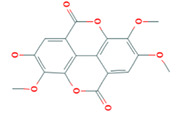
7	Methyl 4,6-di-O-galloyl-beta-D-glucopyranoside	MOL005862	498.43	48.07	0.68	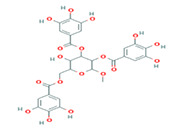
8	Methyl-6-O-galloyl-β-D-glucopyranoside	MOL005864	346.32	44.85	0.29	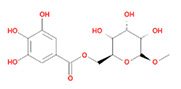
9	Daucostero_qt	MOL005869	414.79	36.91	0.75	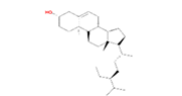
10	Ssauvissimoside R1	MOL005880	696.92	37.39	0.31	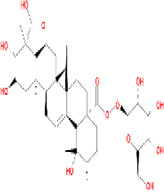
11	Quercetin	MOL000098	302.25	46.43	0.28	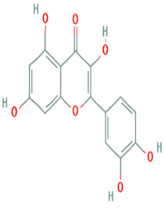
12	(+)-Catechin	MOL000492	290.29	54.83	0.24	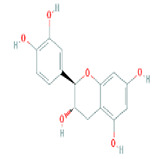
13	Gibberellin	MOL005236	346.41	81.59	0.53	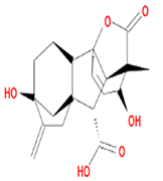
14	Ellagic acid	MOL001002	302.2	43.06	0.43	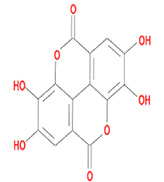

### Active Ingredient–Target Network Construction of *S. officinalis*

**Figure 1:**
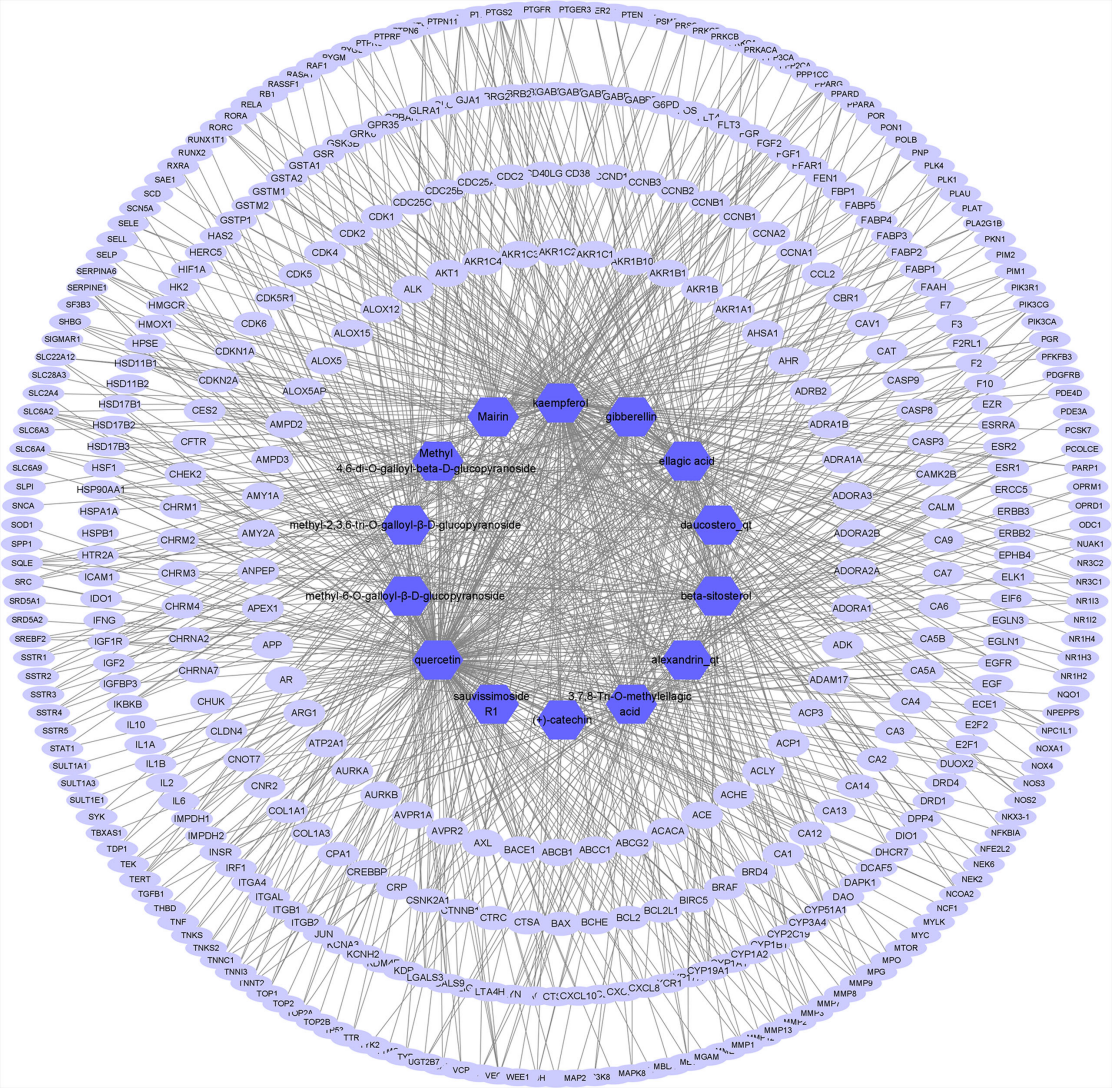
Active ingredient–target network of *S. officinalis*. The network consists of 14 active ingredients and 426 targets. The blue octagons represent active ingredients, and the lilac ellipses represent targets.

The presumed protein targets of the 14 active ingredients were obtained from TCMSP and STP, and a total of 426 targets were found. Then, a compound–target network, consisting of 441 nodes and 935 edges, was constructed to explore the relationship between the ingredients and the targets (**Figure 1**).

### Active Ingredient Target–CRC Target Network Construction

**Figure 2:**
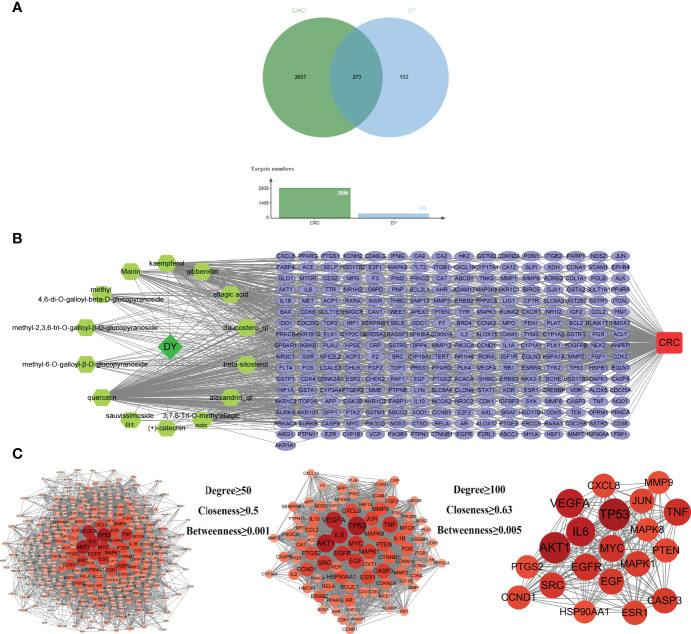
Active ingredient–target–colorectal cancer (CRC) network and protein–protein interaction (PPI) network of common targets. **(A)** The Venn diagram and column diagram of *S. officinalis* targets and CRC targets. **(B)** Active ingredient–target–CRC network. The green octagons represent active ingredients, the lilac ellipses represent targets, and the red rounded rectangle represents CRC. The network consists of 1 CRC disease, 1 *S. officinalis*, 14 active ingredients, and 273 targets. **(C)** The process of the PPI network of *S. officinalis*–target–CRC screening. Circles represent targets. The redder the circle is, the larger the degree value of the target.

By screening GeneCards, OMIM, DrugBank, and TTD databases, 2,930 gene targets related with CRC were obtained. In total, 426 targets of *S. officinalis* were matched to 2,930 targets of CRC, and 273 common targets were found by looking for the intersection of active ingredient targets and CRC targets (**Figure 2A**). An active ingredient–CRC-target network with 289 nodes (1 *S. officinalis* node, 1 CRC node, 14 active ingredient nodes, and 273 common target nodes) and 903 edges was constructed (**Figure 2B**).

### PPI Network Construction of Drug–Disease-Target

The PPI network was obtained by inputting the common targets of *S. officinalis* and CRC into the STRING database, containing 271 nodes and 6,107 edges (**Figure 2C**). According to three network parameters of “degree”, “betweenness”, and “closeness”, 88 major gene targets were screened by the threshold values of degree ≥50, betweenness ≥0.001, and closeness ≥0.5. The firstly screened PPI network contained 88 nodes and 2,261 edges, and the second screening threshold values of degree ≥100, betweenness ≥0.005, and closeness ≥0.63 were used to further screen the 88 major targets. The 20 key gene targets were obtained (**Figure 2C**). The second PPI network was then constructed that included 20 nodes and 190 edges, and the top 5 core targets were TP53, AKT1, IL6, VEGFA, and TNF, respectively.

### GO and KEGG Pathway Enrichment Analyses of Common Targets

**Figure 3:**
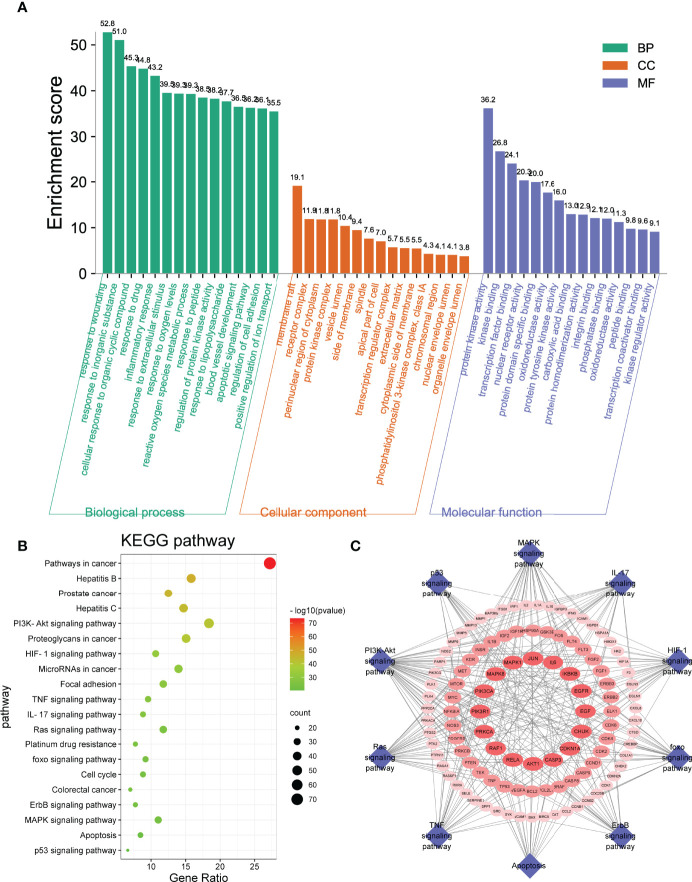
Gene Ontology and Kyoto Encyclopedia of Genes and Genomes enrichment analyses. **(A)** The GO analysis includes the top 15 terms in biological process, cellular component, and molecular function. **(B)** Top 20 enriched pathways of common targets. The color scales mean the different thresholds of adjusted *p*-values, and the sizes of the dots indicate the gene counts of each term. **(C)** Target–pathway network. The blue diamonds represent the pathways, and the red ellipses represent the targets.

The GO and KEGG pathway enrichment analyses of drug–disease–target were executed by a Metascape platform to explore the underlying molecular mechanism of *S. officinalis* against CRC. The GO enrichment results showed that there were 282 biological process (BP), 120 cellular component (CC), and 259 molecular function (MF) terms in total. The top 15 significantly enriched terms of BP, CC, and MF are shown in **Figure 3A**, demonstrating that the targets were related with response to wounding, apoptotic signaling pathway, regulation of cell adhesion (BP), transcription regulator complex, phosphatidylinositol 3-kinase complex (CC), and protein kinase activity, kinase binding, transcription factor binding (MF). These results indicated that *S. officinalis* might regulate cell proliferation, apoptosis, or metastasis through these cellular terms. The KEGG analysis results showed that *S. officinalis* significantly influenced the pathways in cancer, such as PI3K–Akt, HIF-1, TNF, IL-17, Ras signaling pathways, *etc*. (**Figure 3B**). Besides this, the target–pathway network was constructed to further investigate the molecular mechanism of *S. officinalis* anti-CRC (**Figure 3C**). The network consisted of 117 nodes (15 pathways and 102 genes) and 276 edges. AKT1, CASP3, CDKN1A, CHUK, EGF, EGFR, IKBKB, IL6, JUN, MAPK1, MAPK8, PIK3R1, PRKCA, RAF1, and RELA were high-degree gene targets.

### *S. officinalis* Inhibits Cell Proliferation

**Figure 4:**
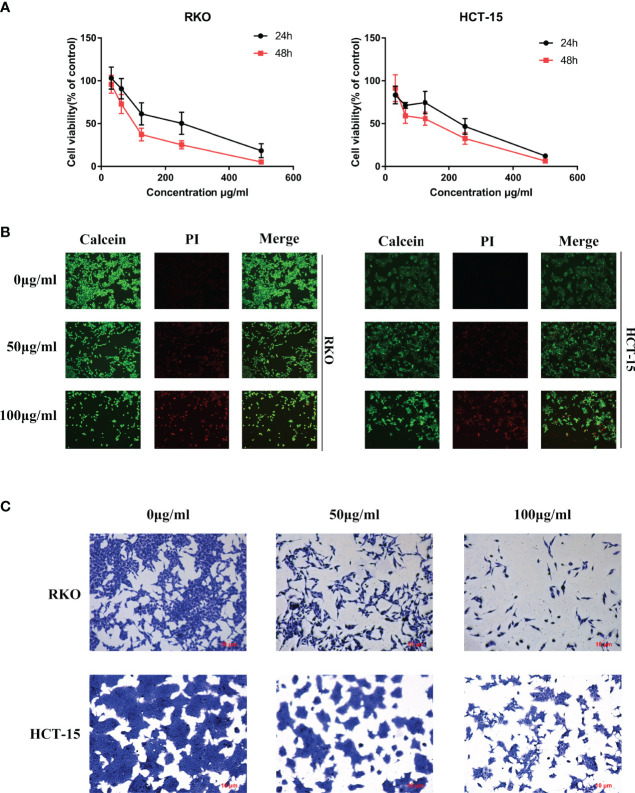
*S. officinalis* inhibits colorectal cancer (CRC) cell proliferation. **(A)** Cells were treated with *S. officinalis* for 24 or 48 h, and 3-(4,5-dimethylthiazol-2-yl)-2,5-diphenyltetrazolium bromide assay was applied to determine the cell viability. The results are expressed as mean ± SD (*n* = 3). **(B)** Cells treated with *S. officinalis* for 48 h were loaded with calcein/propidium iodide buffer, and the cells were observed under a fluorescence microscope (×200). The green fluorescence represents active cells, and the red fluorescence represents dead cells. **(C)** The cellular morphologies of CRC cells with 48 h of *S. officinalis* treatment were observed under a microscope (×200).

To determine the anti-tumor effect of *S. officinalis* as predicted analysis of network pharmacology, the MTT, calcein/PI stain and crystal violet stain assays were performed on CRC cell lines. After 24 or 48 h of treatment, *S. officinalis* evidently inhibited cell proliferation in a dose- and time-dependent manner (**Figure 4A**). The half-maximal inhibitory concentration (IC_50)_ values of *S. officinalis* at 24 h in RKO and HCT-15 cells were 214.8 and 192.2 μg/ml, respectively. The IC_50_ values of *S. officinalis* in RKO and HCT-15 cells at 48 h were 108.2 and 121.2 μg/ml, respectively (**Table 2**). According to the IC_50_ values, the concentrations of 50 and 100 μg/ml were used in the subsequent experiments.

**Table 2:** Half-maximal inhibitory concentration (IC_50_) values of *S. officinalis* on colorectal cancer cells.

	IC_50_ (μg/ml)	
	24 h	48 h
RKO	214.8 ± 6.07	108.2 ± 3.97
HCT-15	192.2 ± 9.15	121.2 ± 2.95

Next, calcein/PI stain assay was applied to verify the effect of *S. officinalis* on cell viability and cytotoxicity. The results showed that *S. officinalis* reduced the percentage of active cells (green fluorescence) and increased the percentage of dead cells (red fluorescence) in a dose-dependent manner after *S. officinalis* treatment for 48 h (**Figure 4B**). The crystal violet stain assay further confirmed that *S. officinalis* evidently reduced cell viability and increased cytotoxicity in a dose-dependent manner (**Figure 4C**). In general, these findings demonstrated that *S. officinalis* markedly inhibited cell proliferation.

### *S. officinalis* Arrests Cell Cycle at G0–G1 Phase

**Figure 5:**
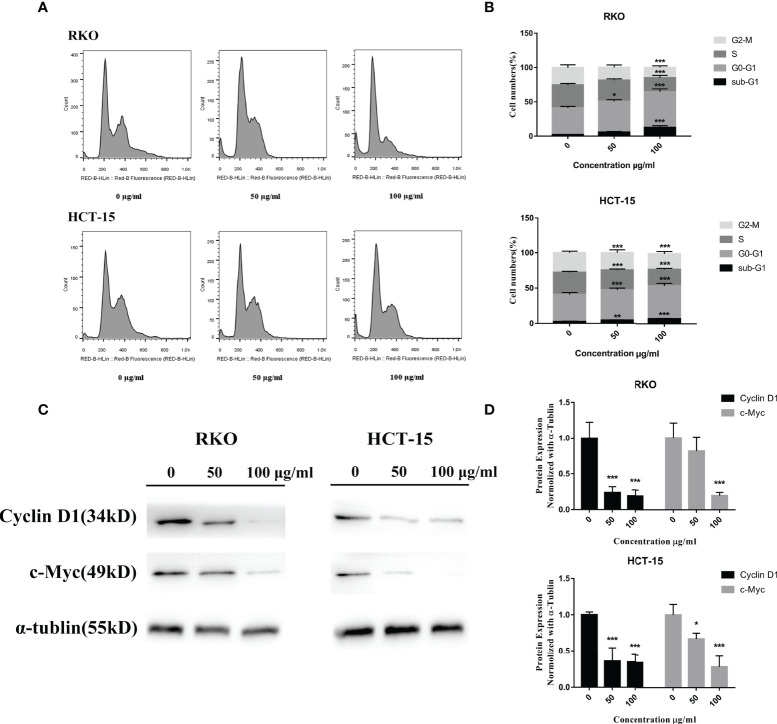
*S. officinalis* arrested the cell cycle at G0–G1 phase. **(A)** The cells were stained by propidium iodide and RNase after 24 h of *S. officinalis* treatment, and the DNA contents were then measured by flow cytometry. **(B)** The histogram shows the percentage of cell numbers in cell cycle phases in the treatment and control groups. The results are expressed as mean ± SD (*n* = 3). *p < 0.05, **p < 0.01, ***p < 0.001, compared with the control group. **(C)** Following the treatment with *S. officinalis* for 24 h, the expression levels of cyclin D1 and c-Myc proteins were determined by western blot and analyzed by α-tubulin. **(D)** The histogram shows the protein expression level in the treatment and control groups. The results are expressed as mean ± SD (*n* = 3). *p < 0.05, **p < 0.01, ***p < 0.001, compared with the control group.

To test the relationship of cell cycle arrest and the anti-proliferative effect of *S. officinalis*, cells were exposed to *S. officinalis* for 24 h (**Figure 5A, B**). The results showed that the phases of sub-G1 were increased, indicating that cells were apoptosis or necrosis. Various cells were arrested in the G0–G1 phase after *S. officinalis* treatment. The proportion of RKO and HCT-15 cells at the G0–G1 phase treated by 100 μg/ml *S. officinalis* for 24 h was altered from 39 and 38.4% to 52.1 and 46.6%, respectively. The expression levels of cyclin D1 and c-Myc proteins, playing a critical role in cell cycle progression, were conducted to further authenticate the cell cycle arrest (**Figures 5C, D**). The results showed that the expression levels of these two proteins were clearly reduced, leading to the cell cycle *via* blocking the transition through G1/S.

### *S. officinalis* Suppresses CRC Cell Migration

**Figure 6:**
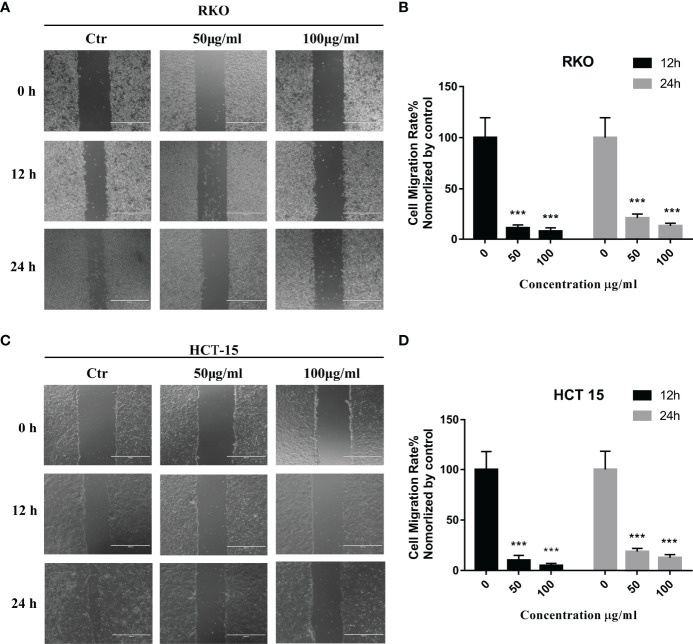
*S. officinalis* suppressed the colorectal cancer cell migration. **(A)** Wound healing of RKO cell with 12 or 24 h of *S. officinalis* treatment was observed under a microscope (×100). **(B)** The histogram shows the cell migration rate in the treatment and control groups. The results are expressed as mean ± SD (*n* = 3). ***p < 0.001, compared with the control group. **(C)** Wound healing of HCT-15 cell with 12 or 24 h of *S. officinalis* treatment was observed under a microscope (×100). **(D)** The histogram shows the cell migration rate in the treatment and control groups. The results are expressed as mean ± SD (*n* = 3). ***p < 0.001, compared with the control group.

The results of network pharmacology showed that *S. officinalis* could inhibit cell migration in addition to the anti-proliferative effect of CRC cells. In order to verify the anti-migrative effect, wound healing assay was performed. Compared with the control group, the cell migration rates of RKO and HCT-15 cells were significantly suppressed after *S. officinalis* treatment in a dose-dependent manner (**Figure 6**). These findings indicated that *S. officinalis* suppressed CRC cell migration.

### Functional Analysis of DEGs of CRC Cells Treated With *S. officinalis*

**Figure 7:**
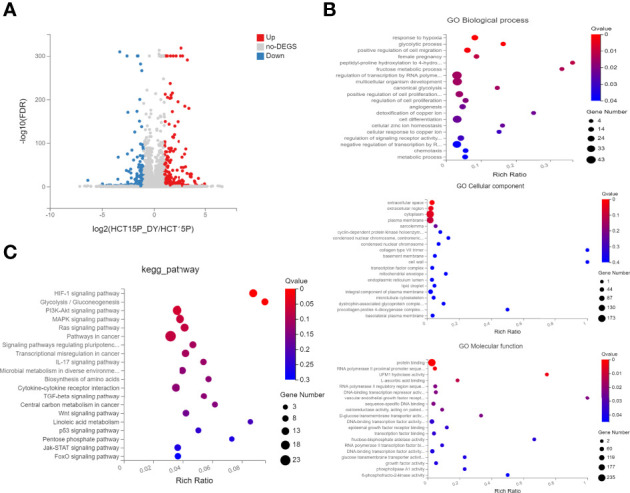
The GO and KEGG enrichment analyses of significantly differentially expressed genes (DEGs). **(A)** Volcano plots of DEGs. The red dots indicate the upregulated mRNAs, and the green dots represent the downregulated mRNAs [|log_2_(FC)| ≥1 and false discovery rate ≤0.001]. **(B)** GO enrichment analysis of DEGs, including the top 15 terms in biological process, cellular component, and molecular function. **(C)** The top 20 enriched pathways of DEGs.

Next, whole transcriptomic sequencing was performed to elucidate the mechanisms, and the DEGs were analyzed. The results showed that 435 mRNAs were identified to be significantly differentially expressed (|log_2_FC| ≥1, FDR ≤0.001) between the *S. officinalis*-treated and control groups (**Figure 7A**). Among these, 236 mRNAs were found to be significantly upregulated and 199 mRNAs were downregulated. Then, GO and KEGG pathway analyses were used to evaluate the molecular processes and biological pathways with DEGs. The GO results showed that the DEGs were enriched in response to hypoxia, positive regulation of cell migration, regulation of cell proliferation, angiogenesis, and cell differentiation (BP), epidermal growth factor receptor binding, DNA-binding transcription repressor activity, *etc*. (MF), and extracellular space and region, cytoplasm and transcription factor complex, *etc*. (CC) (**Figure 7B**). These data implied that *S. officinalis* might inhibit the cell proliferation and migration *via* affecting the above-mentioned molecular processes, which was consistent with the GO results of network pharmacology. The KEGG pathway analysis showed that the most involved signals of DEGs are pathways in cancer, and the top five signals that were significantly affected by *S. officinalis* were the HIF-1, PI3K–Akt, MAPK, Ras, and IL-17 pathways, respectively (**Figure 7C**). Among them, four pathways (HIF-1, PI3K–Akt, Ras, and IL-17 pathways) were the same as in the KEGG analysis of network pharmacology.

### *S. officinalis* Suppresses Pathways Related to Cell Proliferation and Migration

**Figure 8:**
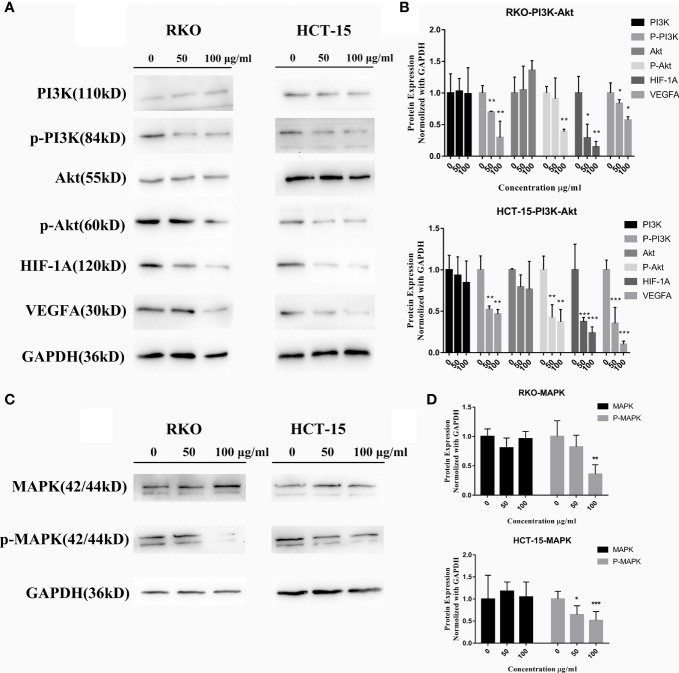
*S. officinalis* suppresses the pathways related to cell proliferation and migration. **(A)** The expression of PI3K, p-PI3K, Akt, p-Akt, HIF-1A, and VEGFA proteins in RKO and HCT-15 cells after *S. officinalis* treatment for 24 h. **(B)** The histogram shows the protein expression level normalized with GAPDH in the treatment and control groups. The results are expressed as mean ± SD (*n* = 3). *p < 0.05, **p < 0.01, ***p < 0.001, compared with the control group. **(C)** Expression of MAPK (p44 and p42) and p-MAPK proteins. **(D)** The histogram shows the protein expression normalized with GAPDH in the treatment and control groups. The results are expressed as mean ± SD (*n* = 3). *p < 0.05, **p < 0.01, ***p < 0.001, compared with the control group.

The KEGG analyses of network and transcriptomic sequencing suggested that the PI3K–Akt, HIF-1, and MAPK pathways may be highly related to the molecular mechanism of *S. officinalis* anti-CRC *via* regulating cell proliferation and migration, so the expression levels of PI3K, p-PI3K, Akt, p-Akt, HIF-1A, VEGFA, MAPK (p44 and p42), and p-MAPK proteins were determined by western blot. The data showed that the expression of p-PI3K, p-Akt, HIF-1A, VEGFA, and p-MAPK proteins was remarkably downregulated in a dose-dependent manner after 24 h of *S. officinalis* treatment (**Figure 8**), while the expression levels of PI3K, Akt, and MAPK proteins were not significantly changed. Taken together, *S. officinalis* inhibited cell proliferation and migration *via* regulating the Akt, HIF-1, and MAPK pathways.

## Discussion

CRC is one of the most malignant cancers globally. The mechanism of CRC development is a complex multistage process, involving multiple targets and pathways. *S. officinalis* has been reported to have therapeutic effects in various cancers. However, there are less studies on CRC, especially on the molecular mechanism. In this study, the network pharmacology strategy combined with whole transcriptomic sequencing approach was applied to explore the characteristics of “multiple ingredients, multiple targets, and multiple pathways” related to *S. officinalis* against CRC.

A total of 14 compounds of *S. officinalis* were identified as active ingredients according to the criteria of OB >30% and DL >0.18. Some of them have been reported to exhibit anti-cancer effects. Mairin or betulinic acid possessed anti-tumor activities against melanoma, osteosarcoma, glioma, leukemia, as well as lung, colon, breast, prostate, hepatocellular, bladder, stomach, pancreatic, ovarian, and cervical carcinoma (13). Beta-sitosterol, a well-known phytosterol contained in many plants, inhibited prostatic, colon, and liver cancers and myeloma *via* inducing apoptosis (14). Kaempferol, one of the most encountered aglycone flavonoids, inhibited various cancer cells by inducing apoptosis, cell cycle arrest, and downregulation of signaling pathways (15). Catechins exhibited inhibitory effects against tumorigenesis in various studies, such as oral cavity, esophagus, stomach, small intestine, and colon cancers (16). Ellagic acid induced apoptosis *via* suppressing PI3K/Akt pathway activation and Bax expression level, and caspase-3 activation was also observed, leading to cell death (17). All these studies support the prediction of network pharmacology.

Among the top 5 core targets including TP53, AKT1, IL6, VEGFA, and TNF screened out in the PPI network analysis, AKT1, as the predominant Akt, has a critical role in cancer cell growth, survival, and metastasis (18). Enhanced AKT1 expression in cancer cells leads to cell proliferation and cell cycle through various downstream effectors, including cyclin D1, GSK-3, mTOR, *etc*. (19). AKT1 deletion in K-Ras mutant mouse models prevented tumorigenesis in the lungs (20). Selective AKT1 inhibitor A-674563 strengthened cancer cell apoptosis compared to pan-Akt inhibitor MK-2206, which demonstrated that targeting the AKT1 isoform might be more effective than suppressing the Akt isoforms (21). Apart from its cell survival and proliferation roles, AKT1 has also been involved in cell migration and invasion. The silence of AKT1 not only abolished the properties of metastasis but also inhibited the expression of epithelial to mesenchymal transition markers, such as vimentin protein which is related to invasive cancers (22). Vascular endothelial growth factor-A (VEGFA) is an endothelial growth factor as well as a regulator of vascular permeability (23), widely expressed in most malignant cancers (24). Hypoxia-inducible factor (HIF) is a major driver of VEGF expression in cancers. HIF-mediated transcription was observed in aggressive cancer cells and induced VEGFA expression (25). Therefore, the prevention of angiogenesis through the inhibition of AKT1 and VEGFA could be an effective treatment strategy for many types of cancers.

According to the KEGG enrichment results of network pharmacology and transcriptomic sequencing, *S. officinalis* regulated the overlapping pathways in the first five pathways, including the PI3K–Akt, HIF-1, Ras, and IL-17 pathways. The RAS-MAPK pathway has frequently been reported to regulate cancer cell growth, proliferation, apoptosis, and migration (26). In normal cells, Ras is inactive (off-state), while upon extracellular stimuli, Ras is activated. Once Ras is activated, MEK1 and MEK2 are activated and catalyze the activation of the effector ERK1 and ERK2 kinases. ERK1/ERK2 broadly phosphorylate downstream effector targets involved in cellular responses, such as cell proliferation, survival, and angiogenesis (27). A recent study has demonstrated that Ras could activate other pathways including the PI3K pathway, associated with the invasiveness of tumor cells (26). The PI3K–Akt pathway is a critical regulator of cell proliferation and survival (28). Akt protein is considered as the key mediator of the PI3K–Akt pathway. As for cell growth, Akt regulates G1/S phase in the cell cycle through the inactivation of GSK-3, resulting in increased cyclin D1 expression (29). In addition, the PI3K–Akt pathway has positive regulation of the hypoxia-induced factor-1α (HIF-1α) protein expression level. HIF-1α is also an important regulator of VEGF, erythropoietin, and glycolytic enzymes. This regulation is PI3K–Akt inhibition dependent, demonstrating that the PI3K–Akt, HIF-1α, and VEGF targets are interrelated (30). Thus, these complex pathways have been considered as one of the most attractive targets for anticancer agents. Our western blot results, including downregulating the expression of p-PI3K, p-Akt, HIF-1α, VEGFA, and p-MAPK proteins, were consistent with the those from the network pharmacology, demonstrating that *S. officinalis* suppressed cell proliferation, migration, and survival through the PI3K–Akt, HIF-1, and MAPK pathways.

Our findings are similar to those recently reported in hepatocellular carcinoma (6). Our study combined the transcriptomics to analyze the molecular mechanism, which was more systematical at the cellular level (mRNA). In addition, we found more active ingredients of *S. officinalis*, such as ellagic acid and (+)-catechin, which were active compounds in *S. officinalis* with good anticancer activity. In terms of mechanism, we verified the downstream targets affected by the PI3K–Akt pathway, such as HIF-1α, VEGFA, and cyclin D1 proteins related to cell metastasis and cell cycle, revealing the molecular mechanism of *S. officinalis* in a more comprehensive way.

These are some limitations to this study. Firstly, the ingredients of *S. officinalis* which were not found in UPLC-MS should be identified by a more optimal method. Secondly, there are other targets (TP53, TNF, EGF, *etc*.) or pathways (Ras and P53) which may play critical roles in the anti-CRC effect of *S. officinalis*. Further studies should be performed to explore the effect of *S. officinalis* on CRC cells. In addition, there is a lack of *in vivo* study on *S. officinalis*. Despite the limitations, this study provides a scientific basis for *S. officinalis* as a potential therapeutic agent for CRC treatment. This study supports the combination of network pharmacology, transcriptomics, and experimental verification to understand the mechanism of actions of Chinese herbal medicines.

## Conclusion

In conclusion, network pharmacology and transcriptomic sequencing analysis, in combination with *in vitro* studies, have been successfully applied to study the underlying mechanism of *S. officinalis* against CRC cells. Our results demonstrate that *S. officinalis* suppresses the proliferation, survival, and migration of CRC cells through regulating the PI3K–Akt, HIF-1, and MAPK signaling pathways.

## Data Availability Statement

The original contributions presented in the study are publicly available. This data can be found here: https://www.ncbi.nlm.nih.gov/bioproject/, PRJNA796145.

## Author Contributions

WZ designed and performed the overall experiments and prepared the manuscript. SS and CP participated in the *in vitro* experiments. LO studied the network pharmacology analysis. ZF and YZ performed data processing. GL and YY participated in the revision of the manuscript. MY designed the overall study and prepared the manuscript. All authors contributed to the article and approved the submitted version.

## Funding

This work was supported by the National Natural Science Foundation of China (number 81973552) and the Natural Science Foundation of Guangdong Province (2022A1515012056).

## Conflict of Interest

The authors declare that the research was conducted in the absence of any commercial or financial relationships that could be construed as a potential conflict of interest.

## Publisher’s Note

All claims expressed in this article are solely those of the authors and do not necessarily represent those of their affiliated organizations, or those of the publisher, the editors and the reviewers. Any product that may be evaluated in this article, or claim that may be made by its manufacturer, is not guaranteed or endorsed by the publisher.

## References

[1] Siegel RL Miller KD Fuchs HE Jemal A. Cancer Statistic. CA Cancer J Clin (2021) 71:7–33. doi: 10.3322/caac.21654 33433946

[2] Xi Y Xu P. Global Colorectal Cancer Burden in 2020 and Projections to 2040. Transl Oncol (2021) 14:101174. doi: 10.1016/j.tranon.2021.101174 34243011 PMC8273208

[3] Liu Y Yang S Wang K Lu J Bao X Wang R. Cellular Senescence and Cancer: Focusing on Traditional Chinese Medicine and Natural Products. Cell Prolif (2020) 53:e12894. doi: 10.1111/cpr.12894 32881115 PMC7574878

[4] Lachowicz S Oszmianski J Rapak A Ochmian I. Profile and Content of Phenolic Compounds in Leaves, Flowers, Roots, and Stalks of Sanguisorba Officinalis L. Determined With the LC-DAD-ESI-QTOF-MS/MS Analysis and Their *In Vitro* Antioxidant, Antidiabetic, Antiproliferative Potency. Pharmaceuticals (Basel) (2020) 13:191. doi: 10.3390/ph13080191 PMC7464974 32806688

[5] Zhao Z He X Zhang Q Wei X Huang L Fang JC. Traditional Uses, Chemical Constituents and Biological Activities of Plants From the Genus Sanguisorba L. Am J Chin Med (2017) 45:199–224. doi: 10.1142/S0192415X17500136 28249548

[6] Jiang N Li H Sun Y Zeng J Yang F Kantawong F. Network Pharmacology and Pharmacological Evaluation Reveals the Mechanism of the Sanguisorba Officinalis in Suppressing Hepatocellular Carcinoma. Front Pharmacol (2021) 12:618522. doi: 10.3389/fphar.2021.618522 33746755 PMC7969657

[7] Liu MP Liao M Dai C Chen JF Yang CJ Liu M. Sanguisorba Officinalis L Synergistically Enhanced 5-Fluorouracil Cytotoxicity in Colorectal Cancer Cells by Promoting a Reactive Oxygen Species-Mediated, Mitochondria-Caspase-Dependent Apoptotic Pathway. Sci Rep (2016) 6:34245. doi: 10.1038/srep34245 27671231 PMC5037464

[8] Yuan H Ma Q Cui H Liu G Zhao X Li W. How Can Synergism of Traditional Medicines Benefit From Network Pharmacology? Molecules (2017) 22:1135. doi: 10.3390/molecules22071135 PMC6152294 28686181

[9] Yang X Kui L Tang M Li D Wei K Chen W. High-Throughput Transcriptome Profiling in Drug and Biomarker Discovery. Front Genet (2020) 11:19. doi: 10.3389/fgene.2020.00019 32117438 PMC7013098

[10] Jang E Inn KS Jang YP Lee KT Lee JH. Phytotherapeutic Activities of Sanguisorba Officinalis and Its Chemical Constituents: A Review. Am J Chin Med (2018) 46:299–318. doi: 10.1142/S0192415X18500155 29433389

[11] Zhang W Peng C Shen X Yuan Y Zhang W Yang C. A Bioactive Compound From Sanguisorba Officinalis L. Inhibits Cell Proliferation and Induces Cell Death in 5-Fluorouracil-Sensitive/Resistant Colorectal Cancer Cells. Molecules (2021) 26:3843. doi: 10.3390/molecules26133843 34202548 PMC8270258

[12] Zhang W Peng C Yan J Chen P Jiang C Sang S. Sanguisorba Officinalis L. Suppresses 5-Fluorouracil-Sensitive and-Resistant Colorectal Cancer Growth and Metastasis *via* Inhibition of the Wnt/beta-Catenin Pathway. Phytomedicine (2022) 94:153844. doi: 10.1016/j.phymed.2021.153844 34785413

[13] Zhang DM Xu HG Wang L Li YJ Sun PH Wu XM. Betulinic Acid and Its Derivatives as Potential Antitumor Agents. Med Res Rev (2015) 35:1127–55. doi: 10.1002/med.21353 26032847

[14] Sook SH Lee HJ Kim JH Sohn EJ Jung JH Kim B. Reactive Oxygen Species-Mediated Activation of AMP-Activated Protein Kinase and C-Jun N-Terminal Kinase Plays a Critical Role in Beta-Sitosterol-Induced Apoptosis in Multiple Myeloma U266 Cells. Phytother Res (2014) 28:387–94. doi: 10.1002/ptr.4999 23640957

[15] Imran M Salehi B Sharifi-Rad J Aslam Gondal T Saeed F Imran A. Kaempferol: A Key Emphasis to Its Anticancer Potential. Molecules (2019) 24:2277. doi: 10.3390/molecules24122277 PMC6631472 31248102

[16] Yang CS Wang H. Cancer Preventive Activities of Tea Catechins. Molecules (2016) 21:1679. doi: 10.3390/molecules21121679 PMC6273642 27941682

[17] Ceci C Lacal PM Tentori L De Martino MG Miano R Graziani G. Experimental Evidence of the Antitumor, Antimetastatic and Antiangiogenic Activity of Ellagic Acid. Nutrients Nov (2018) 14:10. doi: 10.3390/nu10111756 PMC6266224 30441769

[18] Somanath PR Chen J Byzova TV. Akt1 Is Necessary for the Vascular Maturation and Angiogenesis During Cutaneous Wound Healing. Angiogenesis (2008) 11:277–88. doi: 10.1007/s10456-008-9111-7 PMC2677211 18415691

[19] Alwhaibi A Verma A Adil MS Somanath PR. The Unconventional Role of Akt1 in the Advanced Cancers and in Diabetes-Promoted Carcinogenesis. Pharmacol Res (2019) 145:104270. doi: 10.1016/j.phrs.2019.104270 31078742 PMC6659399

[20] Hollander MC Maier CR Hobbs EA Ashmore AR Linnoila RI Dennis PA. Akt1 Deletion Prevents Lung Tumorigenesis by Mutant K-Ras. Oncogene (2011) 30:1812–21. doi: 10.1038/onc.2010.556 PMC4133779 21242979

[21] Franks SE Briah R Jones RA Moorehead RA. Unique Roles of Akt1 and Akt2 in IGF-IR Mediated Lung Tumorigenesis. Oncotarget (2016) 7:3297–316. doi: 10.18632/oncotarget.6489 PMC4823107 26654940

[22] Zhu QS Rosenblatt K Huang KL Lahat G Brobey R Bolshakov S. Vimentin Is a Novel AKT1 Target Mediating Motility and Invasion. Oncogene (2011) 30:457–70. doi: 10.1038/onc.2010.421 PMC3010301 20856200

[23] Claesson-Welsh L Welsh M. VEGFA and Tumour Angiogenesis. J Intern Med (2013) 273:114–27. doi: 10.1111/joim.12019 23216836

[24] Matsumoto K Ema M. Roles of VEGF-A Signalling in Development, Regeneration, and Tumours. J Biochem (2014) 156:1–10. doi: 10.1093/jb/mvu031 24839295

[25] Mimeault M Batra SK. Hypoxia-Inducing Factors as Master Regulators of Stemness Properties and Altered Metabolism of Cancer- and Metastasis-Initiating Cells. J Cell Mol Med (2013) 17:30–54. doi: 10.1111/jcmm.12004 23301832 PMC3560853

[26] Santarpia L Lippman SM El-Naggar AK. Targeting the MAPK-RAS-RAF Signaling Pathway in Cancer Therapy. Expert Opin Ther Targets (2012) 16:103–19. doi: 10.1517/14728222.2011.645805 PMC3457779 22239440

[27] Mebratu Y Tesfaigzi Y. How ERK1/2 Activation Controls Cell Proliferation and Cell Death: Is Subcellular Localization the Answer? Cell Cycle (2009) 8:1168–75. doi: 10.4161/cc.8.8.8147 PMC2728430 19282669

[28] Porta C Paglino C Mosca A. Targeting PI3K/Akt/mTOR Signaling in Cancer. Front Oncol (2014) 4:64. doi: 10.3389/fonc.2014.00064 24782981 PMC3995050

[29] Liang J Slingerland JM. Multiple Roles of the PI3K/PKB (Akt) Pathway in Cell Cycle Progression. Cell Cycle (2003) 2:339–45. doi: 10.4161/cc.2.4.433 12851486

[30] Li L Qu Y Mao M Xiong Y Mu D. The Involvement of Phosphoinositid 3-Kinase/Akt Pathway in the Activation of Hypoxia-Inducible Factor-1alpha in the Developing Rat Brain After Hypoxia-Ischemia. Brain Res (2008) 1197:152–8. doi: 10.1016/j.brainres.2007.12.059 18241842

