# Supplementary material for: Network Pharmacology and Transcriptomic Sequencing Analyses Reveal the Molecular Mechanism of Sanguisorba officinalis Against Colorectal Cancer
Source: Front Oncol. 2022 May 12;12:807718. doi: 10.3389/fonc.2022.807718 (PMC9133337; doi:10.3389/fonc.2022.807718)
Supplement: Supplementary file 1 [file DataSheet_1.docx]

**Supplementary**

Figure S1. The TIC diagram of *S. officinalis* in the anion mode. 9: Mairin; 11: methyl-6-O-galloyl-β-D-glucopyranoside; 14: (+)-catechin; 17: methyl 4,6-di-O-galloyl-beta-D-glucopyranoside; 20: 3,7,8-Tri-O-methylellagic acid; 23: ellagic acid; 25: quercetin; 28: methyl-2,3,6-tri-O-galloyl-β-D-glucopyranoside; 30: kaempferol


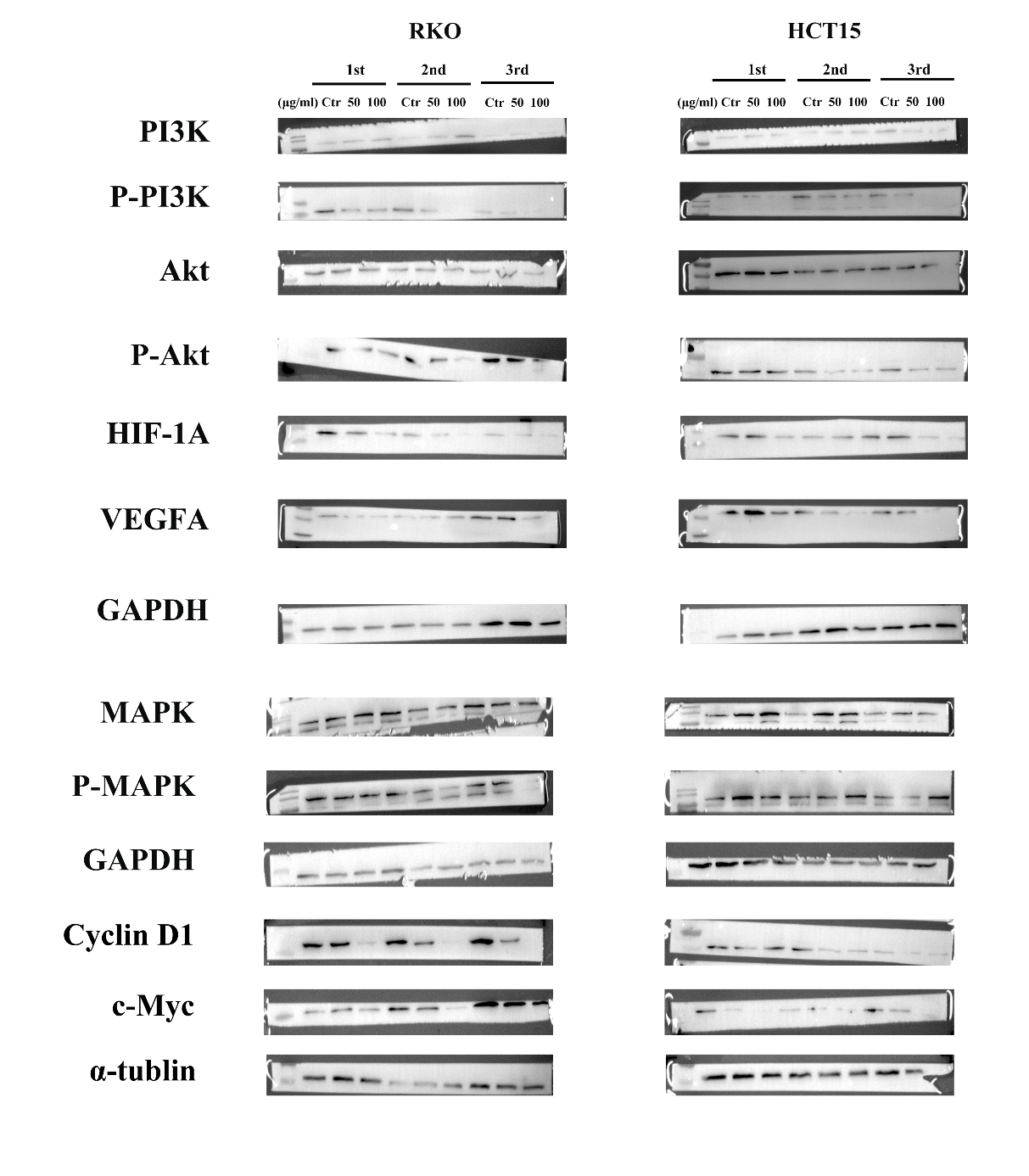
Figure S2. The raw data of the western blotting assays that were repeated triplicates.
